# Optimizing Recovery: Heliox Therapy for Post-extubation Stridor Management

**DOI:** 10.7759/cureus.78740

**Published:** 2025-02-08

**Authors:** Nishant Allena, Shalini Penikilapate, Sai Allu, Trupti Vakde

**Affiliations:** 1 Pulmonary Medicine, BronxCare Health System, Bronx, USA; 2 Internal Medicine, BronxCare Health System, Bronx, USA; 3 Medicine, BronxCare Health System, Bronx, USA; 4 Pulmonary and Critical Care, BronxCare Health System, Bronx, USA

**Keywords:** extubation failure, heliox, post-extubation stridor, traumatic intubation, vocal cord paralysis

## Abstract

Post-extubation stridor poses a significant challenge in critical care settings, often necessitating prompt intervention to prevent respiratory compromise and potential reintubation. This case report details the successful management of post-extubation stridor in a 55-year-old female patient with a complex medical history, using heliox therapy. Heliox, a gas mixture of helium and oxygen, has emerged as a novel therapeutic option in such scenarios, owing to its ability to reduce airway resistance and improve gas flow dynamics.

Following the patient's elective intubation for MRI imaging, she developed hoarseness, loud breathing, and stridor upon extubation, indicative of subglottic edema and bilateral vocal cord paralysis. Despite initial treatment with conventional modalities yielding minimal improvement, heliox therapy (70%/30%) with supplemental oxygen was initiated, resulting in significant alleviation of symptoms. Subsequent maintenance therapy with corticosteroids and bronchodilators further facilitated the resolution of respiratory distress.

This case underscores the pivotal role of heliox therapy as an effective adjunct in managing post-extubation stridor, offering rapid relief and potentially obviating the need for reintubation. Moreover, it highlights the importance of innovative therapeutic approaches in optimizing outcomes for patients with respiratory distress in critical care settings. However, further research is warranted to elucidate the optimal utilization criteria and long-term efficacy of heliox therapy in this context.

## Introduction

The management of post-extubation stridor, a common complication following the removal of an endotracheal tube [1], continues to challenge healthcare professionals. It often leads to respiratory distress and potential reintubation, significantly impacting patient outcomes. This case report presents a 55-year-old female patient with a complex medical history who developed post-extubation stridor. The report highlights the successful use of Heliox in managing the patient's post-extubation stridor, offering insights into the potential benefits of this novel approach.

## Case presentation

A 55-year-old female, with a medical history significant for hypertension, cerebrovascular accident/transient ischemic attack without residual deficits, polysubstance abuse, and asthma, presented with acute left upper and lower extremity weakness, accompanied by nausea and a single episode of vomiting. Upon arrival in the emergency department (ED), the patient was hypertensive with a blood pressure of 231/147 mmHg, tachycardic to 118 beats per minute, and a respiratory rate of 18 with oxygen saturation maintained at 98% on room air. Although oriented to time and place, the patient displayed agitation and non-compliance with commands. An NIH Stroke Scale (NIHSS) score of 1 was documented, and a physical examination revealed unremarkable findings in the chest and abdominal regions.

Initial laboratory investigations demonstrated a white blood cell count of 6.1 K/uL (4.8-10.8 K/uL), hemoglobin/hematocrit of 11.8 g/dL/36.9% (12-16 g/dL/42-51%), and platelet count of 250,000 K/uL (150-400 K/uL). The basic metabolic panel revealed a sodium level of 145 mEq/L (135-145 mEq/L), potassium of 4.2 mEq/L (3.5-5 mEq/L), blood urea nitrogen (BUN) of 23 mg/dL (6-20 mg/dL), creatinine of 1.2 mg/dL (0.5-1.5 mg/dL), and creatine kinase (CK) of 215 units/L (20-200 units/L), and these have been tabulated in Table 1.

**Table 1:** Initial labs on admission

Parameter	Result	Reference value
White blood cell count	6.1 K/uL	4.8-10.8 K/uL
Hemoglobin/hematocrit	11.8 g/dL/36.9%	12-26 g/dL/42-51%
Platelet	250,000 K/uL	150-400 K/uL
Sodium	145 mEq/l	135-145 mEq/L
Potassium	4.2 mEq/l	3.5-5 mEq/L
Blood urea nitrogen	23 mg/dL	6-20 mg/dL
Creatinine	1.2 mg/dL	0.5-1.5 mg/dL
Creatine kinase (CK)	215 units/L	20-200 units/L

**Figure 1:**
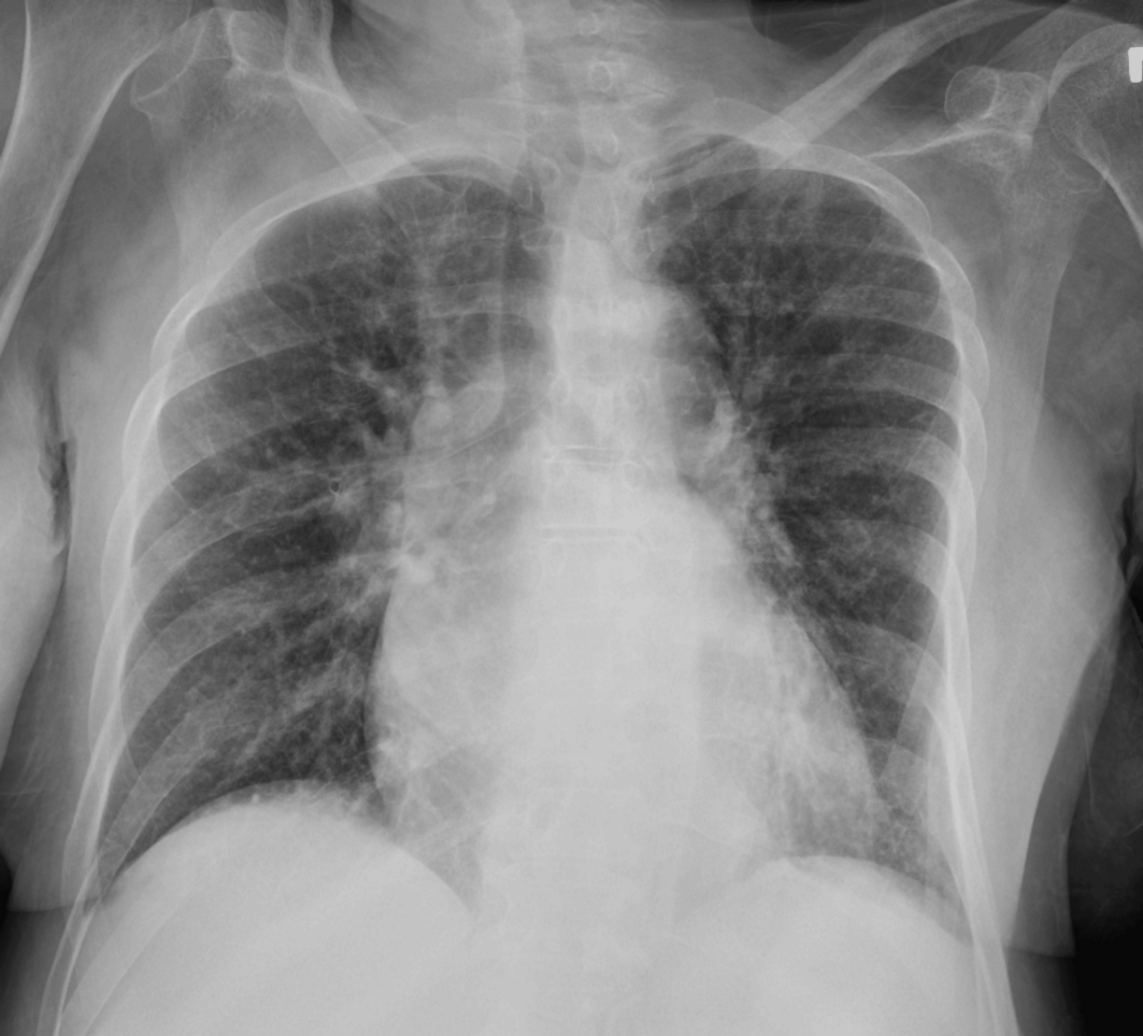
X-ray (antero-posterior view) showing no pulmonary pathology

**Figure 2:**
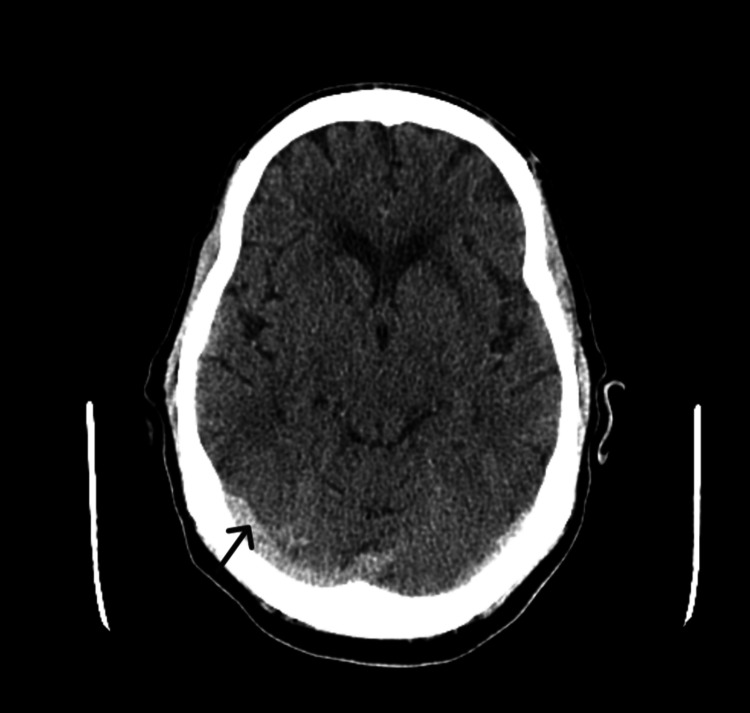
CT of the head without contrast showing right parietal paramedian extra-axial density suspicious for either hemorrhage or neoplasm (arrow)

Ethanol levels were negligible, while urine toxicology was positive for cocaine. Chest X-ray showed no acute pulmonary abnormalities (Figure 1), and a computed tomography of the head (CTH) revealed a right parietal paramedian extra-axial density, raising suspicion for either hemorrhage or neoplasm (Figure 2). CT angiography of the head and neck was negative for vascular anomalies.

Due to the possibility of hypertensive emergency and hemorrhagic stroke, the patient was admitted to the intensive care unit (ICU). She presented with drowsiness interspersed with agitation, prompting the initiation of nicardipine infusion for blood pressure control. Psychiatric consultation was sought for the management of agitation, leading to the initiation of antipsychotic medications. Neurology recommended an MRI of the brain to further evaluate the parietal paramedian density, which was deferred due to the patient's agitation. Despite attempts at sedation, the patient remained agitated, necessitating elective intubation for the MRI.

**Figure 3:**
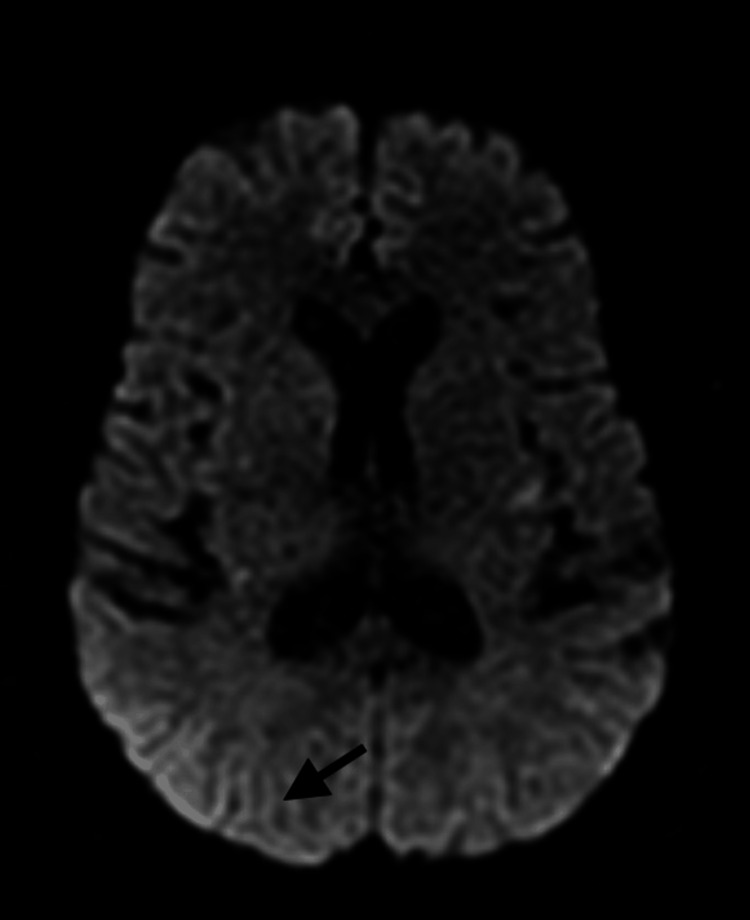
MRI brain showing multiple bilateral acute cerebral infarcts and a 0.9 cm acute intra-axial hemorrhage in the right parieto-occipital region (arrow)

Subsequent MRI revealed multiple bilateral acute cerebral infarcts and a 0.9 cm acute intra-axial hemorrhage in the right parieto-occipital region (Figure 3).

**Figure 4:**
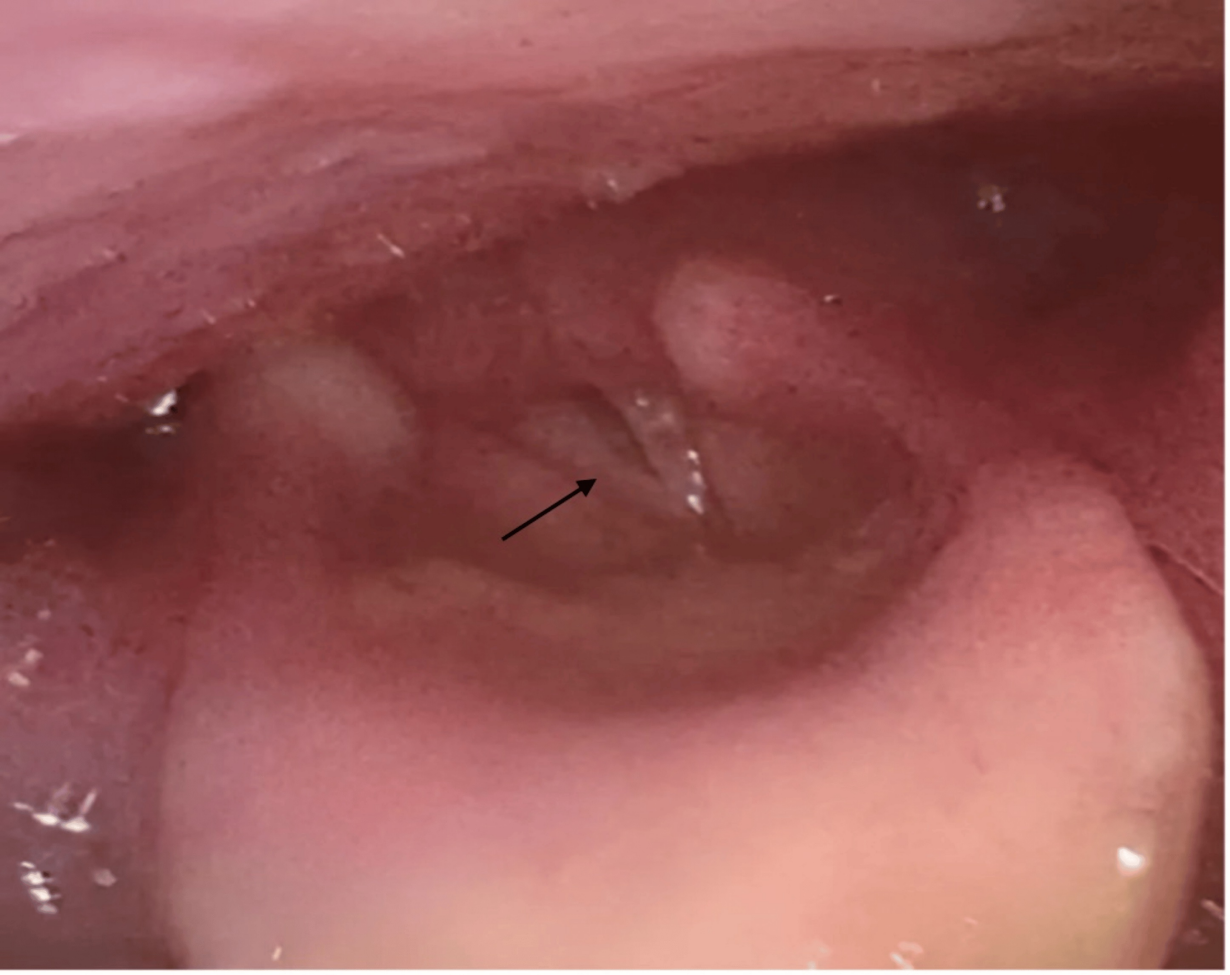
Laryngoscopy imaging showing minimal glottic opening, bilateral vocal cord paralysis, and subglottic edema (arrow)

Following extubation, the patient developed hoarseness, loud breathing, and stridor. Fiberoptic laryngoscopy identified edema and inflammation of the subglottic region, along with bilateral vocal cord paralysis (Figure 4), which was presumed to be due to traumatic intubation.

Initial treatment with IV methylprednisolone (125 mg), inhaled racemic epinephrine (0.5 ml of 2.25% solution), and albuterol-ipratropium nebulization (3 ml) provided minimal benefit. Consequently, heliox therapy (70%/30%) with supplemental oxygen at 10 liters/minute through an aerosol mask was initiated, resulting in significant improvement within 30 minutes.

Maintenance therapy included IV methylprednisolone (62.5 mg every eight hours), racemic epinephrine (0.5 ml of 2.25% solution every six hours), and albuterol-ipratropium nebulization (3 ml) every six hours. One day later, the patient reported marked improvement in hoarseness and was started on a pureed diet. Heliox therapy was discontinued, and oxygen supplementation continued through the nasal cannula. Two days later, the patient's respiratory status improved, and she was transferred to the pulmonary floor. IV methylprednisolone was tapered to oral steroids, and nebulization with (albuterol and ipratropium) and inhaled racemic epinephrine continued every six hours over a period of five days. She was discharged home three days later, demonstrating satisfactory respiratory status and overall improvement in clinical condition.

## Discussion

The use of heliox, a mixture of helium and oxygen, post-extubation presents a novel approach to managing respiratory distress in critically ill patients. This discussion aims to elucidate the rationale, benefits, limitations, and evidence supporting the utilization of heliox in this clinical context. Post-extubation respiratory distress is a common occurrence in critically ill patients, often stemming from underlying lung pathology or prolonged intubation.

Heliox, with its unique physical properties, offers several theoretical advantages in this scenario [2]. By reducing airway resistance and improving gas flow dynamics, heliox may alleviate respiratory distress, enhance patient comfort, and potentially prevent the need for reintubation. Heliox’s lower density leads to decreased airway resistance, particularly beneficial in patients with obstructive lung diseases, thus reducing the work of breathing. Furthermore, the laminar flow facilitated by heliox enhances gas exchange and decreases turbulent airflow, potentially improving oxygenation and ventilation [3]. Jaber et al. [4] demonstrated in a study that heliox therapy post-extubation improved respiratory distress in critically ill patients. Patients often experience reduced dyspnea and improved comfort with heliox therapy, which may improve their tolerance to post-extubation nasal cannula support. Consequently, effective respiratory support provided by heliox may mitigate the need for reintubation, thereby reducing associated complications and healthcare costs [5].

Despite these theoretical benefits, the evidence supporting heliox use post-extubation is primarily derived from small-scale studies and observational data, lacking robust randomized controlled trials. Identifying the ideal patient population for heliox therapy post-extubation remains challenging, with further research needed to delineate specific criteria for optimal utilization [6]. Additionally, heliox administration requires specialized equipment and expertise, which may not be universally available in all healthcare settings, and its use may incur additional costs and resource utilization. While generally well-tolerated, heliox therapy carries potential risks such as barotrauma and fire hazards, necessitating close monitoring and vigilant safety measures. Heliox's role in critical care continues to be explored, necessitating further research to solidify its clinical application and benefits. Table 2 summarizes case reports highlighting the use of heliox with various delivery systems for the management of post-extubation stridor.

**Table 2:** Summary of studies describing the use of heliox in post-extubation stridor BiPAP: bilevel positive airway pressure

Primary cause of indication	Days on ventilator	Mode of delivery of heliox	Case report
Laryngeal edema post-extubation	11 days	BiPAP	Punj et al. [7]
Laryngeal edema post-extubation	7 days	BiPAP	Punj et al. [7]
Subglottic mass/subglottic mucous casting	9 days	Unavailable	St-Onge et al. [8]
Upper airway obstruction following extubation	5 days	Venturi mask	Skrinskas et al. [9]
Bilateral vocal cord dysfunction post-extubation	6 hours	Unavailable	Christopher et al. [10]
Upper airway obstruction (narrowed main-stem bronchus)	Few hours	Mechanical ventilator	Lu et al. [11]
Laryngeal edema after Teflon injection of vocal cord	Few hours	Nebulizer	Solomons and Livesey [12]
Laryngeal edema and left vocal cord paralysis	4 days	Helium head-box	Duncan [13]
Post intubation croup	Few hours	Reservoir mask	Duncan [13]

## Conclusions

Heliox therapy post-extubation holds promise as a potential adjunctive treatment in managing respiratory distress in critically ill patients. While its theoretical advantages are compelling, further research, including large-scale randomized controlled trials, is warranted to delineate its efficacy, safety profile, and optimal patient selection criteria.

## References

[1] Abbasi S, Moradi S, Talakoub R, Kashefi P, Koushki AM (2014). Effect of nebulized budesonide in preventing postextubation complications in critically patients: a prospective, randomized, double-blind, placebo-controlled study. Adv Biomed Res.

[2] Johnson JE, Gavin DJ, Adams-Dramiga S (2002). Effects of training with heliox and noninvasive positive pressure ventilation on exercise ability in patients with severe COPD. Chest.

[3] Dieperink W, Knol JA, Boersma HJ, Eindhoven GB, Aarts LP, Goorhuis JF, Nijsten MW (2007). Combination of heliox and CPAP without a ventilator: bench test and clinical observations. Eur J Anaesthesiol.

[4] Jaber S, Carlucci A, Boussarsar M (2001). Helium-oxygen in the postextubation period decreases inspiratory effort. Am J Respir Crit Care Med.

[5] Hashemian SM, Fallahian F (2014). The use of heliox in critical care. Int J Crit Illn Inj Sci.

[6] Austan F, Polise M (2002). Management of respiratory failure with noninvasive positive pressure ventilation and heliox adjunct. Heart Lung.

[7] Punj P, Nattanmai P, George P, Newey CR (2017). Successful extubation using heliox BiPAP in two patients with postextubation stridor. Case Rep Pulmonol.

[8] St-Onge M, Di Fabio JM, Lazar N (2012). Post-extubation upper airway obstruction: an interesting case. Can J Anaesth.

[9] Skrinskas GJ, Hyland RH, Hutcheon MA (1983). Using helium-oxygen mixtures in the management of acute upper airway obstruction. Can Med Assoc J.

[10] Christopher K, Arbelaez C, Yodice PC (2002). Bilateral vocal cord dysfunction complicating short-term intubation and the utility of heliox. Respiration.

[11] Lu TS, Ohmura A, Wong KC, Hodges MR (1976). Helium-oxygen in treatment of upper airway obstruction. Anesthesiology.

[12] Solomons NB, Livesey JR (1990). Acute upper airway obstruction following Teflon injection of a vocal cord; the value of nebulized adrenaline and a helium/oxygen mixture in its management. J Laryngol Otol.

[13] Duncan PG (1979). Efficacy of helium-oxygen mixtures in the management of severe viral and post-intubation croup. Can Anaesth Soc J.

